# Efficacy and safety of PEG-rhG-CSF in preventing chemoradiotherapy-induced neutropenia in patients with locally advanced cervical cancer

**DOI:** 10.17305/bjbms.2022.7859

**Published:** 2023-03-16

**Authors:** Weiwei Li, Mohan Dong, Shigao Huang, Liu Shi, Hua Yang, Ying Zhang, Jie Gong, Mei Shi, Lichun Wei, Lina Zhao

**Affiliations:** 1 Department of Radiation Oncology, The First Affiliated Hospital, Air Force Medical University, Xi'an, China; 2 Department of Medical Education, The First Affiliated Hospital, Air Force Medical University, Xi'an, China

**Keywords:** Cervical cancer, pegylated recombinant human granulocyte colony-stimulating factor (PEG-rhG-CSF), concurrent chemoradiotherapy (CCRT), radical chemoradiotherapy, neutropenia

## Abstract

The standard of care for locally advanced cervical cancer is concurrent chemoradiotherapy, which is associated with significant toxicity, especially hematologic toxicity. To evaluate the efficacy and safety of pegylated recombinant human granulocyte colony-stimulating factor (PEG-rhG-CSF) in preventing neutropenia during radical chemoradiotherapy for cervical cancer, 40 patients receiving prophylaxis from February 2018 to July 2019 were randomly divided into two arms in a 1:1 ratio. Patients in the study arm (*N* ═ 21) received PEG-rhG-CSF, while patients in the control arm (*N* ═ 19) received short-acting rhG-CSF. The primary endpoint was the incidence of grade 3–4 neutropenia, and the secondary endpoints were the incidence of febrile neutropenia, chemotherapy delay, and radiotherapy interruption. In addition, dynamic changes in absolute neutrophil count during radical chemoradiotherapy and adverse events were compared between the two groups. There were 0 and 4 cycles of grade 3–4 neutropenia in the PEG-rhG-CSF and rhG-CSF groups, respectively. The incidence of neutropenia of all grades was lower in patients on PEG-rhG-CSF than that on rhG-CSF [24.05% (19/79) vs. 56.94% (41/72); *p* < 0.001]. No patient developed neutropenic fever. The lowest values of neutropenia during chemoradiotherapy cycles were 2.73 ± 1.02 and 1.91 ± 0.79 × 10 ^9^/mL in the PEG-rhG-CSF and rhG-CSF groups, respectively (*p* < 0.001). In the PEG-rhG-CSF and rhG-CSF groups, 0 and 8 (11.11%) cycles of chemotherapy were delayed due to neutropenia, respectively (*p* ═ 0.01). There was no delay of radiotherapy by more than one week in either group. Prophylactic use of PEG-rhG-CSF during chemoradiotherapy for cervical cancer can effectively prevent neutropenia and associated adverse events. PEG-rhG-CSF may be an effective strategy to provide uninterrupted radical chemoradiotherapy for cervical cancer.

## Introduction

Cervical cancer is one of the most common gynecologic malignancies, with approximately 130,000 new diagnosed cases in China each year [1]. Patients with cervical cancer usually present in the locally advanced stages [2]. The standard of care for locally advanced cervical cancer (LACC) is concurrent chemoradiotherapy (CCRT) [3]. However, in patients with lymph node metastasis, large primary tumor size, and adenocarcinoma pathologic type [4, 5], CCRT with cisplatin was reported to be unsatisfactory and led to five-year survival rates of 40% [6]. Therefore, new concurrent treatment options need to be explored. The combination of cisplatin and docetaxel has the highest overall response rate of 85.7% in cervical cancer [7]. However, CCRT with both drugs is associated with significant toxicity, particularly hematologic toxicity [8], which can impact the delivery of full doses of CCRT.

Recombinant human granulocyte colony-stimulating factor (rhG-CSF) is used for supportive care and safe administration of chemotherapy. rhG-CSF mobilizes neutrophil progenitor cells to reduce the severity of neutropenia and the risk of febrile neutropenia (FN) by at least 50% [9]. Both the American Society of Clinical Oncology [10] and the European Society of Medical Oncology [9] recommend the routine use of prophylactic rhG-CSF based on the risk of developing FN. The patients receiving the cisplatin–docetaxel combination regimen were assigned to the intermediate group (10%–20% risk) to have FN. A published phase III randomized clinical trial named CONVERT suggested that G-CSF use in the context of CCRT did not increase the toxicities and was beneficial for treatment completion [11]. Therefore, the consensus of the Chinese Association for Therapeutic Radiation Oncologists recommended the application of rhG-CSF during CCRT [12].

However, the short half-life rhG-CSF requires daily administration [13]. Pegylated rhG-CSF (PEG-rhG-CSF) is a long-acting and self-regulating stimulating factor that has a plasma half-life of 47 h. It has been administered with one dose for each cycle of chemotherapy to prevent neutropenia [14]. The effectiveness and safety of the two drugs have been confirmed by clinical studies [15, 16]. However, the prophylactic efficacy of PEG-rhG-CSF during radical chemoradiotherapy for pelvic tumors, such as cervical cancer, was seldom investigated. Therefore, we performed a randomized trial to compare the differences in safety and efficacy between PEG-rhG-CSF and rhG-CSF during chemoradiotherapy in patients with LACC.

**Figure 1.**
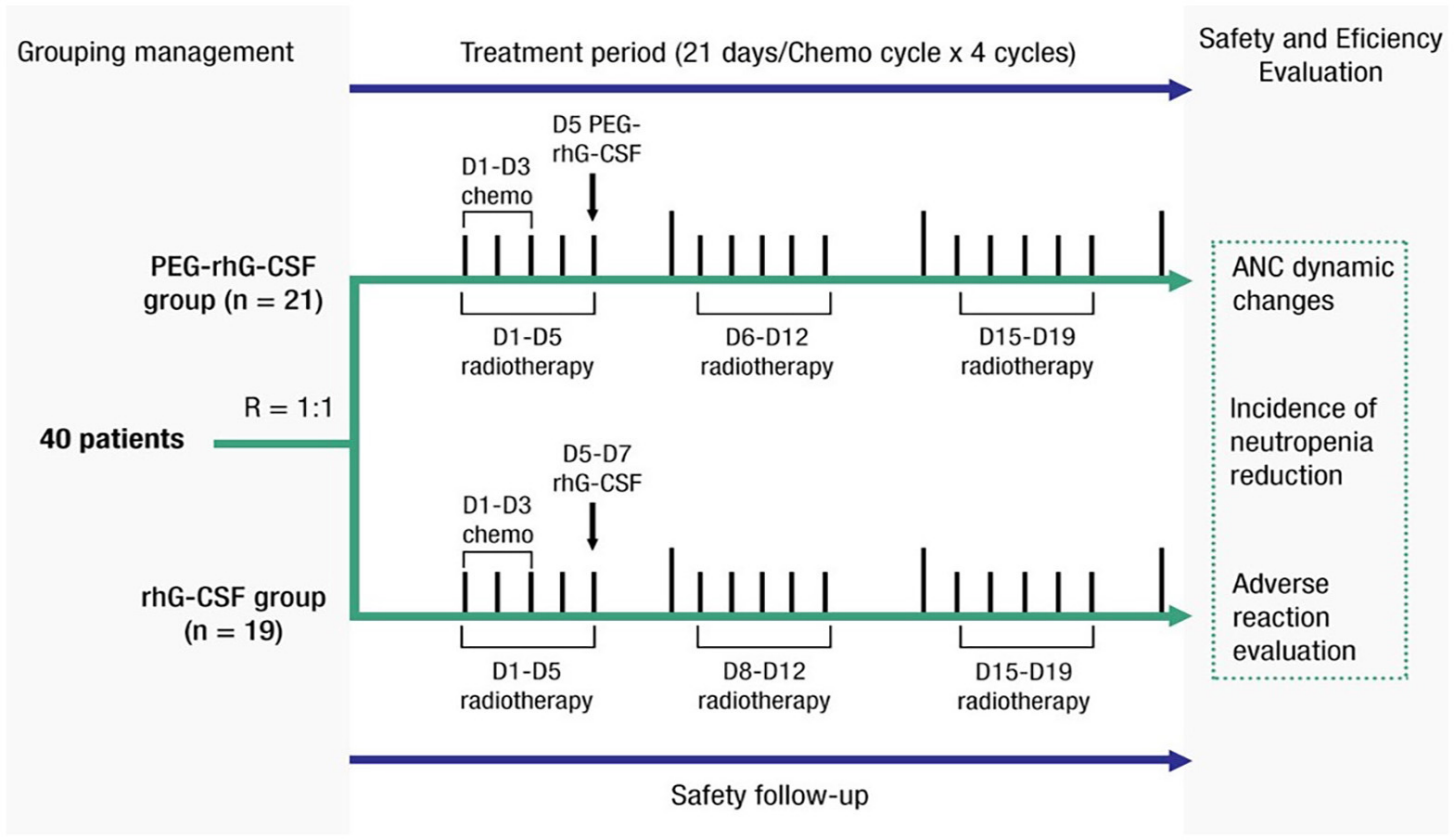
**Schematic diagram of the treatment process**. PEG-rhG-CSF: Pegylated recombinant human granulocyte colony-stimulating factor; rhG-CSF: Recombinant human granulocyte colony-stimulating factor; ANC: Absolute neutrophil count.

## Materials and methods

### Study population and data collection

The trial was a single-center, open-label, randomized study at the First Affiliated Hospital of Air Force Medical University (Xi’an, China) and was registered in ClinicalTrials.gov (NCT03206684). Patients were eligible for inclusion if they had cervical cancer with the 2009 International Federation of Gynecology and Obstetrics (FIGO) IB–IVA stage and were intended to receive radical chemoradiotherapy from February 2018 to July 2019. Eligible patients were also required to be aged ≥18 years, have a Karnofsky performance score of ≥70, and have consented to receive radical chemoradiotherapy regimen with docetaxel and cisplatin. The key exclusion criteria included the presence of (1) other active malignancies or brain metastases, (2) existing infection that is difficult to control, or (3) allergy to PEG-rhG-CSF and rhG-CSF or other biologic products derived from genetically engineered *Escherichia coli*. A full list of the inclusion and exclusion criteria is given in the protocol (https://clinicaltrials.gov/). All patients provided informed consent to participate. The eligible patients were randomly divided at a 1:1 ratio into either the PEG-rhG-CSF group as the study arm or the rhG-CSF group as control. The study design diagram is shown in Figure 1.

### Procedure and treatment

In the study group, PEG-rhG-CSF was given subcutaneously at a dose of 6 mg for patients weighing ≥45 kg or 3 mg for those weighing <45 kg after 48 h of completing one chemotherapy cycle. In the control arm, rhG-CSF was injected subcutaneously at 48 h after the end of chemotherapy at a dose of 300 µg for those weighing ≥45 kg or 150 µg for those weighing <45 kg once a day for three consecutive days. In both groups, if the white blood cell count (WBC) was less than 2 × 10^9^/L or the absolute neutrophil count (ANC) was less than 0.5 × 10^9^/L, rhG-CSF for salvage treatment was administered at a dose of 150–300 µg once a day until a WBC of 10 × 10^9^/L or an ANC of 1.5 × 10^9^/L was reached. Both PEG-rhG-CSF and rhG-CSF were provided for free by the China Shiyao Pharmaceutical Group.

All patients underwent three-dimensional conformal or intensity-modulated radiotherapy of the clinical target at 50 Gy in 25 daily fractions and two cycles of concurrent chemotherapy, followed by an intracavitary brachytherapy boost using a high dose rate technique and two cycles of adjuvant chemotherapy. The metastatic lymph nodes were treated with a simultaneous integrated boost regimen of 62.5 Gy in 25 fractions. Chemotherapy was given once every 21 days and comprised a dual-agent regimen of intravenous docetaxel at 60–75 mg/m^2^ on day 1 and intravenous cisplatin at 20–25 mg/m^2^ on days 1–3. Chemotherapy and radiotherapy were withheld for grade 4 neutropenia, thrombocytopenia, FN, or persistent (i.e., >24 h) grade 3 or 4 gastrointestinal toxicity.

**Table 1:** Basic characteristics of the patients

**Characteristic**	**PEG-rhG-CSF (*N* ═ 21)**	**rhG-CSF (*N* ═ 19)**	***p* value**	**Total (*N* ═ 40)**
Age, median (range)	50 (32–65)	50 (42–68)	0.693	50 (32–68)
KPS score, mean (SD)			0.632	
70	0	1		1
80	13	10		23
90	8	8		16
Pathologic type, *n*				
Squamous cell carcinoma	21	19	–	40
2009 FIGO staging, *n*			0.071	
Ib	2	3		5
IIA	6	1		7
IIB	7	13		20
IIIA	3	0		3
IIIB	2	2		4
IVA	1	0		1
Routine blood, mean (SD)				
Leukocyte (10^9^/L)	7.58 (2.90)	7.53 (4.14)	0.511	7.56 (3.44)
Neutrophils (10^9^/L)	5.84 (2.24)	6.27 (3.89)	0.708	6.03 (3.04)
Hemoglobin (g/L)	115.50 (18.24)	111.23 (17.56)	0.529	113.59 (17.75)
Platelets (10^9^/L)	264.94 (75.91)	274.92 (100.85)	0.763	269.41 (86.44)
External pelvic irradiation time (d), mean (SD)	36.14 (2.97)	37.53 (3.63)	0.361	36.80 (3.33)

PEG-rhG-CSF: Pegylated recombinant human granulocyte colony-stimulating factor; rhG-CSF: Recombinant human granulocyte colony-stimulating factor; FIGO: International Federation of Gynecology and Obstetrics.

### Assessments

Blood test was done once a week or according to physician decision, whereas physical examination and evaluation for toxicity were repeated weekly during treatment. Neutropenia, FN, and safety evaluation were graded and evaluated according to the Common Terminology Criteria for Adverse Events (CTCAE 5.0) of the World Health Organization. Chemotherapy or radiotherapy delay was defined as suspension of the schedule of treatment for more than one week.

### Ethical statement

The trial was a single-center, open-label, randomized study at the First Affiliated Hospital of Air Force Medical University (Xi’an, China) and was registered in ClinicalTrials.gov (NCT03206684). The study protocol was following the Guidelines of Helsinki, reviewed and approved by the Ethics Committee of the First Affiliated Hospital, Air Force Medical University (KY20172040-1). All patients provided informed consent to participate.

### Statistical analysis

The present study intended to reduce the incidence of grade 1–4 neutropenia to 30% in all chemotherapy cycles by the prophylactic use of PEG-rhG-CSF, based on a probability of 65% for the event [17]. Allowing for the loss of 10% of chemotherapy cycles and using a one-tailed alternative hypothesis with an *α* of 0.005 and a *β* of 0.20, the required sample size was 160 cycles.

SPSS19.0 statistical software (SPSS Inc., Chicago, IL, USA) was used for data analysis. Demographic and baseline data were statistically described and compared between the study and control arms. The incidence of the efficacy measures and safety events was statistically described, and 95% confidence intervals were calculated. Student’s t-test, Chi-square test, and Fisher’s exact test were used to compare the differences between the arms. *p* < 0.05 denoted significance.

## Results

### Basic characteristics of the patients

During the study period, 40 patients with LACC were enrolled and randomly assigned to the PEG-rhG-CSF (*n* ═ 21) or rhG-CSF (*n* ═ 19) group. The entire cohort of patients had a median age of 50 years (range, 32–68 years). The patients with FIGO stage IIB–IVA accounted for 70% of the whole cohort. The two arms were balanced in terms of neutrophils, leukocytes, hemoglobin, and platelet count at baseline (Table 1). The mean duration of pelvic irradiation was 36.14 ± 2.97 days in the PEG-rhG-CSF arm and 37.53 ± 3.33 days in the rhG-CSF arm.

### PEG-rhG-CSF reduced the incidence of neutropenia and improved treatment compliance

A total of 151 chemotherapy cycles, including 79 cycles in the PEG-rhG-CSF arm and 72 cycles in the rhG-CSF arm, were used to evaluate safety and efficacy. The incidence of grade 1–4 neutropenia in the PEG-rhG-CSF and rhG-CSF groups was 24.05% and 56.94%, respectively. The incidence of grade 3–4 neutropenia was significantly lower in the PEG-rhG-CSF group than in the rhG-CSF group [0 (0%) vs. 4 (5.56%) cycles; *p* ═ 0.046] (Table 2).

**Table 2:** Incidence of neutropenia and related events in the two arms *N* (%)

**Variables**	**PEG-rhG-CSF**	**rhG-CSF**	***P***
*Grade 3-4 neutropenia (%)*			
CACT (cycles ═ 151)	0/79 (0)	4/72 (5.56)	0.106
Concurrent chemotherapy (cycles ═ 80)	0/42 (0)	2/38 (5.26)	0.430
Adjuvant chemotherapy (cycles ═ 71)	0/37 (0)	2/34 (5.88)	0.436
*Grade 1-4 neutropenia (%)*			
CACT (cycles ═ 151)	19/79 (24.05)	41/72 (56.94)	<0.001*
Concurrent chemotherapy (cycles ═ 80)	12/42 (28.57)	25/38 (65.79)	0.001*
Adjuvant chemotherapy (cycles ═ 71)	7/37 (18.92)	16/34 (47.06)	0.011
*ANC minimum, mean (SD)*			
CACT (cycles ═ 151)	2.73 (1.02)	1.91 (0.79)	<0.001*
Concurrent chemotherapy (cycles ═ 80)	2.65 (0.86)	1.89 (0.74)	<0.001*
Adjuvant chemotherapy (cycles ═ 71)	2.87 (1.22)	1.93 (0.87)	0.0011*
FN (cycles ═ 151)	0	0	–
Chemotherapy delay (%) (cycles ═ 151) Delay time >1 week	0/79 (0)	8/72 (11.11%)	0.01
Salvage treatment with rhG-CSF (151)	0	21/72 (29.17%)	<0.001*
Chemotherapy dose adjustment (cycles ═ 151)	0	0	–
Interruption of radiotherapy (>1 week)	0	0	–

* indicates statistically significant difference (*p* < 0.05). PEG-rhG-CSF: Pegylated recombinant human granulocyte colony-stimulating factor; rhG-CSF: Recombinant human granulocyte colony-stimulating factor; ANC: Absolute neutrophil count; FN: Febrile neutropenia; CACT: Concurrent and adjuvant chemotherapy.

The lowest value of ANC throughout treatment was 2.73± 1.02 and 1.91 ± 0.79 × 10^9^/L in the PEG-rhG-CSF and rhG-CSF arm (*p* ═ 0.004), respectively. Salvage treatment was needed during 21 (29.17%) chemotherapy cycles in the rhG-CSF group but was not needed in the PEG-rhG-CSF group. The incidence of chemotherapy delay was significantly lower in the PEG-rhG-CSF arm than in the rhG-CSF arm [0 (0%) vs. 8 (11.11%); *p* ═ 0.01]. However, in both groups, chemotherapy dose adjustment was not needed and there were no radiotherapy delay and FN.

### Higher level and rapid recovery of neutrophil count in PEG-rhG-CSF arm

**Figure 2.**
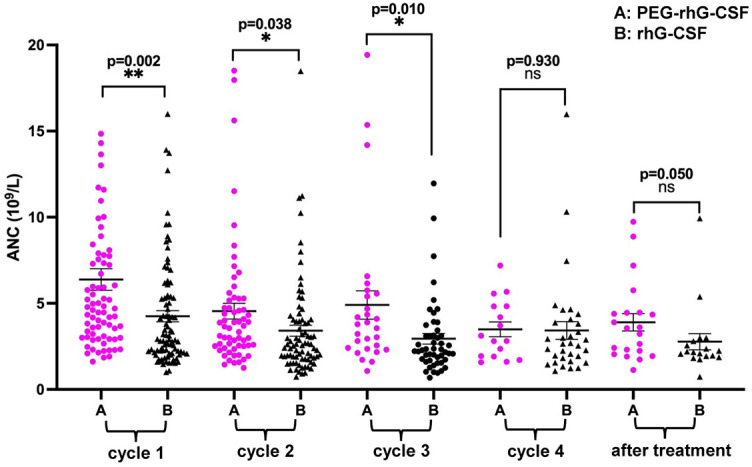
**The mean ANC level during the first until the fourth cycled of chemoradiotherapy.** ANC: Absolute neutrophil count; PEG-rhG-CSF: Pegylated recombinant human granulocyte colony-stimulating factor; rhG-CSF: Recombinant human granulocyte colony-stimulating factor.

The mean ANC level was higher in the PEG-rhG-CSF arm than in the rhG-CSF arm during each period of chemoradiotherapy (Figure 2). In the PEG-rhG-CSF group, the level of ANC was the lowest during the second chemotherapy cycle but rapidly improved during the third chemotherapy cycle after the end of pelvic irradiation. On the other hand, in the rhG-CSF group, the ANC level recovered slowly and at lower levels during the second and third cycles of chemotherapy. The ANC level was not significantly different between the two groups during the fourth chemotherapy cycle (*p* ═ 0.93) and at one month after the end of chemoradiotherapy (*p* ═ 0.05).

### Safety evaluation

Among the adverse reactions encountered in the PEG-rhG-CSF and rhG-CSF groups, digestive tract events, bone marrow suppression, and skin reaction were the most frequent. Among the 40 patients in this study, there were no severe adverse events related with PEG-rhG-CSF or rhG-CSF (Table 3).

**Table 3:** Adverse reactions

**Adverse events**	**PEG-rhG-CSF *N* ═ 21 (%)**		**rhG-CSF *N* ═ 19 (%)**		***p* value**		**Total (*N* ═ 40)**	
	**Grade 1/2**	**Grade 3/4**	**Grade 1/2**	**Grade 3/4**	**Grade 1/2**	**Grade 3/4**	**Grade 1/2**	**Grade 3/4**
Decreased WBC count	11 (52.38)	8 (38.1)	6 (31.58)	12 (63.16)	0.184	0.113	17 (42.5)	20 (50)
Decreased platelet count	15 (71.43)	2 (9.52)	8 (42.11)	1 (5.26)	0.109	0.609	23 (57.5)	3 (7.5)
Anemia	15 (71.43)	6 28.57)	14 (73.68)	5 (26.32)	0.873	0.873	29 (72.5)	11 (27.5)
Upper gastrointestinal symptoms	17 (80.9)	0	15 (78.9)	0	0.874	–	32 (80)	0
Lower gastrointestinal symptoms	20 (95.2)	0	17 (89.5)	0	0.596	–	37 (92.5)	0
Urinary system symptoms	8 (38.1)	0	10 (52.6)	0	0.356	–	18 (45)	0
Skin reaction	9 (42.9)	0	11 (57.9)	0	0.527	–	20 (50)	0

PEG-rhG-CSF: Pegylated recombinant human granulocyte colony-stimulating factor; rhG-CSF: Recombinant human granulocyte colony-stimulating factor; WBC: White blood cell count; –: Not applicable.

## Discussion

In this study, the prophylactic use of PEG-rhG-CSF after every chemotherapy cycle effectively reduced the incidence of grade 1–4 neutropenia and delay of chemotherapy in patients with cervical cancer treated by cisplatin and docetaxel. The use of PEG-rhG-CSF resulted in high levels and fast recovery of neutrophils and low incidence of grade 1–4 neutropenia. There were no severe adverse effects of PEG-rhG-CSF treatment.

Since 1999, CCRT with cisplatin has become the standard of care for patients receiving radical therapy for LACC. However, in patients with poor prognostic factors, such as lymph node metastasis, large primary tumor size, adenocarcinoma pathologic type, and FIGO stage III–IVA, concurrent chemotherapy using single-agent cisplatin cannot significantly improve the survival rate [4, 5]. Therefore, new therapeutic options for these patients with refractory tumors need to be explored. Myelosuppression is an important factor that affects a smooth course of treatment (i.e., radiotherapy alone, chemotherapy, and CCRT) and the prognosis of patients with cervical cancer. The incidence of myelosuppression varies among treatment regimes. For example, the reported incidence rate of grade 1–4 neutropenia with radiotherapy alone, single-agent cisplatin concurrent chemotherapy, and dual-agent chemoradiotherapy was 10.8%, 30.2%, and 82%, respectively [17–19].

In the past, rhG-CSF was used to prevent and treat leukopenia and FN caused by chemotherapy. Koensgen et al. [20] observed the occurrence of severe hematologic toxicity after dual-agent CCRT for LACC. In the present study, the group that received rhG-CSF developed grade 3–4 neutropenia in 5.56%, required salvage treatment with short-acting rhG-CSF during 29.17% of the treatment cycles. In addition, patients had poor compliance to the daily injection of rhG-CSF; this made it difficult to ensure a high ANC level. In other studies, the therapeutic effect of rhG-CSF and survival of patients was altered, because the standard dose and timing of radiotherapy and chemotherapy could not be guaranteed [15, 21, 22]. In the present study, patients who received prophylactic short-acting rhG-CSF developed grade 1–4 neutropenia in 63.24% of the chemotherapy cycles. Moreover, despite prophylactic and therapeutic use of rhG-CSF, 11.11% of the chemotherapy cycles were delayed by more than one week. Therefore, a more efficient drug is needed to ensure the continuity of radiotherapy and chemotherapy.

The plasma half-life of PEG-rhG-CSF is extended to 47 h, and several clinical studies have confirmed that one dose per chemotherapy cycle can effectively prevent neutropenia [14, 23]. In 2017, Liu et al. [24] compared the difference in efficacy between prophylactic use of PEG-rhG-CSF and therapeutic use of rhG-CSF in 163 patients who received CCRT for esophageal cancer and lung cancer. In the prevention and treatment arms, the incidence of hospitalization related with neutropenia was 4.44% and 14.62%, respectively, and the incidence of chemotherapy delay or dose reduction was 5% and 17.69%, respectively. In that study, the protective effect was better with the prophylactic use of PEG-rhG-CSF than with the therapeutic use of rhG-CSF (*p* < 0.05).

In the present study, there was no grade 3–4 neutropenia in patients who received prophylactic PEG-rhG-CSF, and the incidence of grade 1–4 neutropenia was significantly lower in the PEG-rhG-CSF arm (19/79, 24.05%) than in the rhG-CSF arm (41/72, 56.94%). Moreover, the incidence of delay in chemotherapy was significantly lower in the PEG-rhG-CSF arm (0/79, 0%) than in the rhG-CSF arm (8/72, 11.11%). The results of this study showed that compared with multiple doses of short-acting rhG-CSF, one prophylactic administration of PEG-rhG-CSF per chemotherapy cycle resulted in better bone marrow protection. The consensus of Chinese experts on the use of pegylated granulocyte stimulating factor during CCRT does not recommend the use of PEG-rhG-CSF during the weekly chemotherapy regimen of CCRT [12]. Moreover, there is a lack of relevant studies on whether PEG-rhG-CSF can maintain a relatively high level of WBC and neutrophils during uninterrupted radiotherapy, especially in the phase of dual-agent concurrent chemotherapy for cervical cancer.

In this study, we recorded the dynamic changes in neutrophils during chemoradiotherapy and found a relatively high level and rapid increase of the ANC after the end of pelvic irradiation in the PEG-rhG-CSF arm. These results were similar to those in the study by Zou et al. [25], although the control group in the latter study did not receive any prophylactic drug. The high level and rapid recovery of neutrophils were presumed to be associated with the low incidence of treatment delay in the PEG-rhG-CSF arm.

### Limitations

Some limitations of this study were the small number of included patients and the single institute design. However, the total number of chemotherapy cycles met the statistical requirements, because each patient received four cycles of chemotherapy. In addition, the efficacy of rhG-CSF use for three times per cycle in the control group could be a little weak for the prevention of neutropenia. In 2003, Green et al. [26] reported the good results with rhG-CSF use for 10–11 times per cycle; however, patient compliance was poor. Lastly, the effects of PEG-rhG-CSF on survival cannot be evaluated because of the small sample size.

## Conclusion

Prophylactic use of PEG-rhG-CSF during chemoradiotherapy for cervical cancer can effectively prevent neutropenia and its related adverse events. PEG-rhG-CSF use may be an effective strategy to guarantee continuous radical chemoradiotherapy for cervical cancer by ensuring the designed time and dose of chemotherapy and an uninterrupted radiotherapy. This result is worth being further proven by a phase III randomized prospective study.
